# POE Immunoassay: Plate-based oligonucleotide electro-chemiluminescent immunoassay for the quantification of nucleic acids in biological matrices

**DOI:** 10.1038/s41598-020-66829-6

**Published:** 2020-06-26

**Authors:** Mai B. Thayer, Sara C. Humphreys, Kyu S. Chung, Julie M. Lade, Kevin D. Cook, Brooke M. Rock

**Affiliations:** Amgen Research, Pharmacokinetics & Drug Metabolism, Amgen Inc., South San Francisco, CA US

**Keywords:** Biological techniques, Drug discovery, Molecular biology

## Abstract

Oligonucleotide therapeutics use short interfering RNA (siRNA) or antisense oligonucleotide (ASO) molecules to exploit endogenous systems—neutralizing target RNA to prevent subsequent protein translation. While the potential clinical application is vast, delivery efficiency and extrahepatic targeting is challenging. Bioanalytical assays are important in building understanding of these complex relationships. The literature currently lacks description of robust and sensitive methods to measure siRNA and ASOs in complex biological matrices. Described herein is a non-enzymatic hybridization-based immunoassay that enables quantification of individual siRNA strands (antisense or sense) in serum, urine, bile, and liver and kidney homogenates. Assay utility is also demonstrated in ASOs. The assay improves upon previous works by abolishing enzymatic steps and further incorporating Locked Nucleic Acid (LNA) nucleotide modifications to increase analyte hybridization affinity and improve sensitivity, specificity, and robustness. We report an assay with an ultrasensitive dynamic range of 0.3 to 16,700 pM for siRNA in serum. The assay was submitted to full qualification for accuracy and precision in both serum and tissue matrices and assay performance was assessed with single and mixed analytes. The reliable LNA-hybridization-based approach removes the need for matrix sample extraction, enrichment or amplification steps which may be impeded by more advanced chemical modifications.

## Introduction

Oligonucleotide-based drugs have continued to develop as a unique and effective therapeutic class^1^. Capitalizing on targeting cellular pathways that are not readily druggable via a “programmable” RNA sequence-based mechanism-of-action, several nucleic acid therapies have been approved in the clinic^2,3^. In this manuscript we describe the quantitation of two such drug classes: small interfering ribonucleic acids (siRNA) and antisense oligonucleotides (ASOs). ASOs are short, synthetic, single-stranded oligodeoxynucleotides that use Watson-Crick base pairing to form RNA-DNA hybrid substrates for RNase H, which quickly results in target RNA degradation^4^. Working through a different RNA interference (RNAi) pathway, siRNAs are double-stranded oligonucleotides, typically 21 base pairs in length, that work through the initiation of the multi-protein RNA-induced silencing complex (RISC)^5,6^. The RISC machinery guides the complimentary antisense sequence to its RNA target leading to the cleavage of the transcript^7^. Both ASOs and siRNAs have prospered from synthetic chemistry advances that have improved upon stability, off-target effects, and efficacy^4,8–15^. Pharmacokinetics, tissue targeting, and tissue accumulation are all important considerations for both ASOs and siRNA therapeutics. In this paper, we focus on oligonucleotides conjugated directly to a triantennary N-acetylgalactosamine (GalNAc) ligand, which facilitates delivery to the liver via the hepatocyte-expressing asialoglycoprotein receptor (ASGPR)^16^. The importance of bioanalytics in understanding exposure-response relationships is paramount in the development of oligonucleotide therapeutics.

Highly sensitive and quantitative approaches are essential in characterizing siRNA/ASO pharmacokinetics, especially given its low efficacious dose requirements. While various polymerase-chain reaction (PCR) based^17,18^, size-exclusion chromatography (SEC)^19^, and liquid chromatography-mass spectrometry (LC-MS)^20–23^ methods exist, they are limited by their sensitivities and time-consuming extraction steps. In our previously published Hybridization-Ligation assay, we capitalized upon the unprecedented affinity of Locked Nucleic Acids (LNA) modifications to their target sequences^24–26^. While sensitivity, lack of sample extraction steps, and application to complex matrices was achieved, we encountered challenges due to the batch-to-batch variance of commercial enzymes and additional incubation steps. Seeking to eliminate the enzymatic steps, shorten assay runtimes, and further improve sensitivity, we made modifications to our previously published method.Described herein, we demonstrate the utility of the Plate-Based Oligo Electrochemiluminescent (POE) immunoassay for quantifying the antisense and sense strands of the siRNA duplex both sensitively and specifically across various biological matrices. A tool hypoxanthine-guanine phosphoribosyltranferase (HPRT) siRNA was adapted and used to demonstrate assay performance with *in vivo* efficacy summarized in Supplemental Fig. 3^27^. Our qualification of the method against an siRNA molecule demonstrates acceptable precision and accuracy. In addition to qualifying the assay in serum and tissue homogenate, we demonstrate its applicability to ASOs as well as siRNA molecules in a mixture. Furthermore, we show that the assay is applicable to a variety of preclinical species and multiple matrices.

## Material and Methods

### Design of LNA capture and detection probes

**Figure 1:**
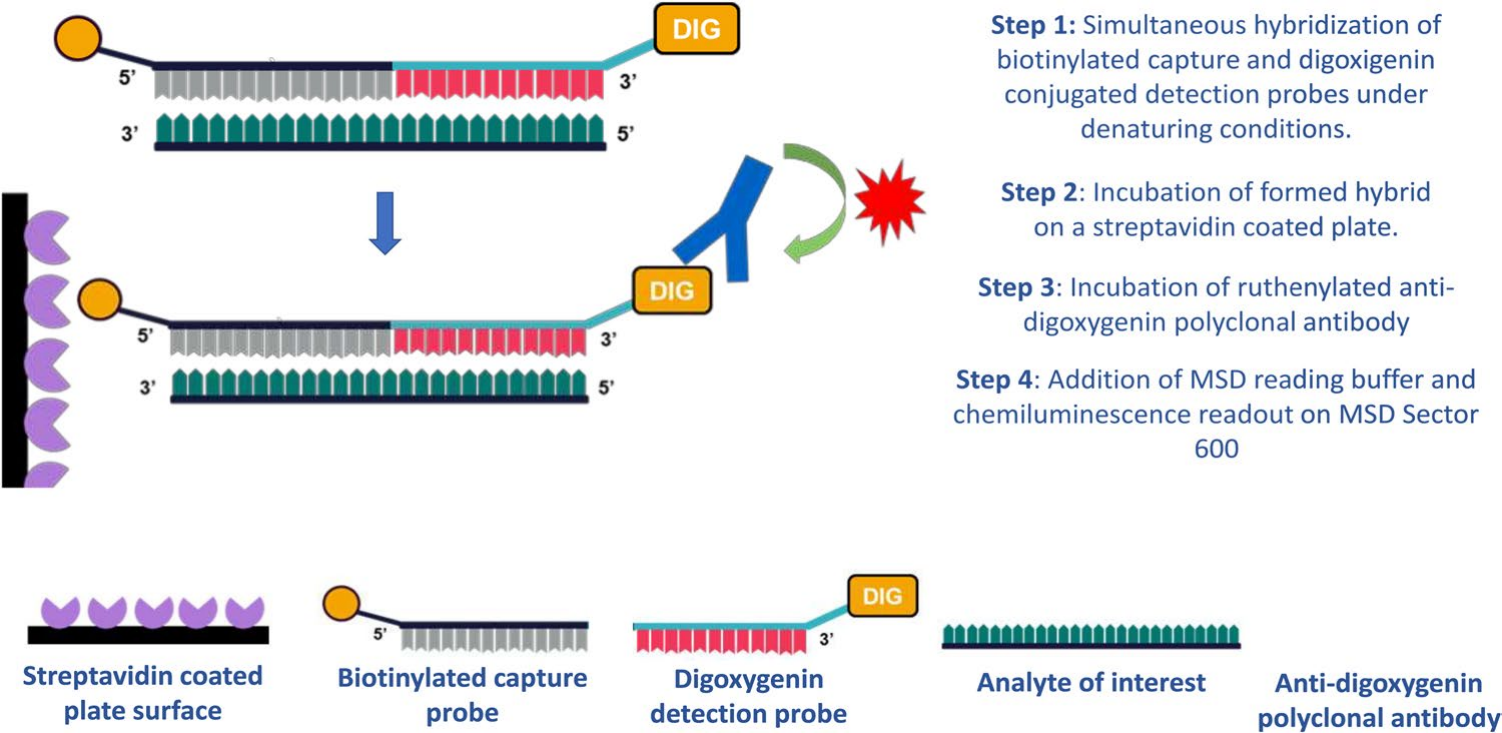
Illustration of the POE immunoassay sandwich with brief overview of assay steps.

Two LNA modified DNA-based oligonucleotide probes of roughly equivalent length were designed to adjacently hybridize to the target analyte sequence via Watson-Crick base-pairing. One probe (11-mer) acts as the capture reagent via a 5′ biotin and the other (10-mer) acts as a detection reagent with a 3′digoxygenin attachment. LNA base incorporations are made to increase the theoretical annealing temperature without creating interfering secondary structure or causing steric constraints. Hybrid stability is sequence based and should be assessed for each individual analyte sequence. A ballpark DNA to RNA probe T_m_ is 60 °C, with assay temperatures run at 40 °C, thermal temperatures which favor hybridization without disrupting protein-protein interactions leading to high assay background. Furthermore, ligand attachment sites are factored into probe designs. Steric hindrance is often observed when the probes orient delivery ligands facing down into the plate surface. Once capture and detection probes are designed, optimal hybridization temperatures are assessed to ensure maximum dynamic range and limited matrix interference. A general schematic with summary assay steps is shown in Fig. 1.

### siRNA and ASO test compound synthesis

Molecules referenced in this manuscript (HPRT.siRNA, x.siRNA, y.siRNA, z.siRNA, and ASO were synthesized using a MerMade 12 Oligonucleotide synthesizer (Bioautomation, Plano TX). The sequence of HPRT.siRNA was adapted from the literature with the sequence of the sense and antisense strands, respectively, as follows: 5′-UCC UAU GAC UGU AGA UUU UA[invAb]-3′ and 5′-AUA AAA UCU ACA GUC AUA GGA UU-3′, with an inverted deoxyribose abasic nucleoside abbreviated as “[invAb]”^24^. For all siRNA molecules included herein, chemical modifications, mainly 2′-*O*-methyl and 2′-deoxy-2′-fluoro ribose modifications, were completely tiled throughout the nucleotide sequence in addition to the incorporation of terminal phosphorothioate backbone linkages to mitigate nuclease activity. Prior to annealing, three GalNAc monosaccharides were conjugated to the 5′ end of the sense strand using a proprietary scaffold and linker design (Amgen Inc., Thousand Oaks, CA) to result in a GalNAc-siRNA. Molecules x.siRNA and z.siRNA comprise two nucleotide overhang structures, whereas HPRT.siRNA and y.siRNA are one-sided blunt-mer designs. The ASO test compound comprises a mixture of deoxy-and ribose nucleotides. A final compound purity of 95% was confirmed for each of the molecules by ion exchange high performance liquid chromatography and the molecular weight was verified using liquid chromatography coupled with mass spectrometry.

### Assay detection reagents

All chemicals and reagents were analytical grade or higher, if not specified. Lyophilized oligonucleotide probes were custom synthesized from Qiagen Inc. (Hilden, Germany). Metabolite simulating 2′OMe modified oligonucleotides were custom synthesized by Integrated DNA Technologies (Iowa, USA). Stock solutions of 100 *µ*M were prepared by reconstitution in molecular grade water. Sheep polyclonal anti-digoxygenin antibody (Roche, Cat. 11222089001) was conjugated with a ruthenium label using the MSD Gold Sulfo-Tag NHS-Ester conjugation kit (Meso Scale Diagnostics, Cat. R31AA-1). The modified DNA sequences of HPRT.siRNA and ASO probes are shown below.

HPRT.siRNA antisense capture probe: 5′-/5Bio/ AAT CCT ATG ACT-3′

HPRT.siRNA sense capture probe: 5′-/5Bio/ TAA AAT CTA CA-3′

HPRT.siRNA antisense detection probe: 5′-GTA GAT TTT AT /3Dig_N/-3′

HPRT.siRNA sense detection probe: 5′-G TCA TAG GA /3Dig_N/-3′

ASO capture probe: 5′-/5Bio/GAA CAA GCA C-3′

ASO detection probe: 5′-CAA CGG AGC A/3Dig_N/-3′

*Underlined base: LNA modification*

*5Bio: Biotin Conjugation with C6 Linker*

*3Dig_N: Digoxygenin (NHS) Ester.*

### Standard curve and sample preparation

Standard curves included in qualification assays involved a targeted dilution range of HPRT.siRNA prepared at 4 to 2,500 ng/mL in 100% matrix. Quality control (QC) spikes were prepared at 10, 800, and 1,250 ng/mL for serum and 5.6, 450, and 1,250 ng/mL in liver tissue homogenate. Matrices for standards, QCs, and unknown sample dilution were obtained from BioIVT (Westbury, NY).

Unknown *in vivo* tissue samples were homogenized in lysis buffer containing 50 mM Tris HCl, 100 nM NaCl, 0.1% Triton X100, and protease inhibitor cocktail (Roche, Cat. 11836170001) to a final concentration of 200 mg/mL. Blood samples collected were incubated for 20 minutes at room temperature, once clotted, whole blood was centrifuged at 14,000 *g* for 15 minutes to isolate serum for exposure measurements. Further dilution series of serum samples were diluted in 100% untreated, pooled serum matrix. Further dilution of tissue samples was done in 10% tissue homogenate (200 mg/mL weight per volume unit) diluted in sample buffer consisting of 10 mM Tris-HCl [pH 8.0] and 1 mM EDTA.

### POE immunoassay procedure

Duplex siRNA or ASO were spiked into tissue homogenate or serum at a designated standard curve range. The standard curves and study samples were diluted 1:10 and 1:50 for siRNA and ASO assays, respectively. Diluted samples were added to a 96-well PCR plate to a final volume of 50 *µ*L. Oligonucleotide sequence specific capture and detection oligonucleotides were prepared in a hybridization buffer consisting of 60 mM Na_2_PO_4_ [pH 7.0, dibasic], 1 M NaCl, 5 mM EDTA, and 0.02% Tween 20. A 2X mixture of probes in hybridization buffer was prepared at 20 nM, and 50 *µ*L of this mixture was added to the PCR plate containing diluted standards and unknowns. The final mixture within the PCR plate resulted in a probe concentration of 10 nM and sample dilution value of 1:20. Sample and probes were hybridized on a thermal cycler under the following conditions: 90 °C for 5 minutes, 40 °C for 30 minutes, and a final hold at 12 °C. After hybridization, 45 *µ*L of samples were transferred to an MSD Gold 96-well Streptavidin SECTOR plate (Meso Scale Diagnostics, LLC., Rockville, MD) in duplicate and were incubated on a shaking platform for 30 minutes. After incubation, the plate was washed with 1X KPL wash solution (SeraCare, Milford, MA). After washing, plates were incubated for 1 hour with 50 *µ*L of 0.5 *µ*g/mL ruthenium labeled anti-digoxygenin antibody in SuperBlock T20 TBS Blocking Buffer (Thermo Fisher Scientific). After a final wash, 150 *µ*L of 1X MSD Read Buffer T (Meso Scale Diagnostics, LLC., Rockville, MD) was added and the plate was read on an MSD Sector S 600 instrument (Meso Scale Diagnostics, LLC., Rockville, MD). A nonlinear regression analysis was performed to calculate the concentrations of reference compound from the signal intensities via interpolation from a calibration curve using 4-parameter logistic (4PL) model (weighting factor = 1/Y^2^) in Watson LIMS 7.5 (Thermo Fisher Scientific, Philadelphia, PA).

### Method qualification

To assess the robustness and precision of the POE immunoassay method, we performed a representative qualification of the antisense strand of HPRT.siRNA in both rat serum and liver tissue homogenate. We evaluated the linearity of the assay with a concentration range of 4 to 2,500 ng/mL pre-20-fold dilution into the assay plate. Both precision and accuracy were determined from a low, mid, and high concentration spike for within-day and between-day parameters. Serum QC spikes for low, mid, and high concentrations corresponded with 10, 800, and 1,250 ng/mL, respectively. Liver tissue homogenate QC spikes for low, mid, and high concentrations corresponded with 5.6, 450, and 1,250 ng/mL, respectively. Due to the high targeted accumulation of GalNAc siRNA in targeting and clearance related tissues^16^, dilutional linearity was also evaluated out to 1000-fold in serum and 10,000-fold in liver tissue homogenate. A high concentration test article stock was used to ensure that the first dilution contained greater than 95% matrix relative.

### Stability of HPRT.siRNA antisense strand

The stability of HPRT.siRNA antisense strand was evaluated with spike-preparations made in both rat serum and liver tissue homogenates at low, middle, and high QC concentrations in 100% matrix. Samples were frozen at −80 °C and defrosted at room temperature for a total of three cycles and their extrapolated values were compared to a freshly prepared calibration curve. Temperature stability was assessed holding prepared QC spikes at room temperature for 4 hours and at −80 °C, referred to as deep freeze (DF), for greater than one week. Temperature stability QC spikes were compared to a freshly prepared calibration curve.

### Specificity and selectivity

To assess the general specificity of the POE probes, synthetic metabolites of the antisense strand of z.siRNA were prepared as previously described and characterized^25^. Standard curves of both parent compound and metabolites were prepared in PBS buffer and the EC_50_ values were compared. Selectivity of the assay was tested in two parts: comparing reactivity when the analyte is varied against a constant probe set, and when an analyte is held constant against a varied probe set combination.

### Assay of siRNAs in a mixture

Linearity of siRNAs in a mixture was also characterized. Test compounds HPRT.siRNA, x.siRNA, y.siRNA, and z.siRNA were prepared in a single calibration curve at equivalent concentrations in rat serum. Samples were run in duplicate and sense and antisense assay linearity was assessed for each respective compound and POE probe pair.

### Animal protocols

Animals were housed in groups at an AAALAC, International accredited facility. Animals were cared for in accordance with the Guide for the Care and Use of Laboratory Animals, 8^th^ Edition. All research protocols were reviewed and approved by the Amgen Institutional Animal Care and Use Committee. C57BL/6 male mice (8 weeks) were acquired from The Jackson Laboratory (Sacramento, CA), and Sprague-Dawley male rats (ten weeks), were acquired from Charles River Laboratories. Animals were housed in individual ventilated caging (IVC) system on an irradiated corncob bedding (Envigo Teklad 7097). Lighting in animal holding rooms was maintained on 12:12 hour light:dark cycle, and the ambient temperature and humidity range was at 68 to 79 F and 30 to 70%, respectively. Animals had ad libitum access to irradiated and pelleted feed (Envigo Teklad Global Rodent Diet-soy protein free extruded 2020X) and reverse-osmosis (RO) chlorinated (0.3 to 0.5 ppm) water via an automatic watering system. Test compound was administered to mice as a SC bolus into the mid-scapular areas at a single 5 mg/kg dose per animal (n = 2). Blood was collected at 0, 0.083, 0.25, 0.5, 1, 2, 4, 8, 24, 48, and 96 hrs post dose. Test compound was administered to rats as a SC bolus into the mid-scapular and mid-dorsal areas for a total of 3 doses, once weekly, for a total dose of 10 mg/kg per animal (n = 5). Blood was collected at 0, 0.083, 2, 6, 14, and 48 hours post first and second dose, with a final collection taken alongside necropsy on day 16. Pharmacokinetic parameters were extrapolated from sense and antisense serum concentrations using WinNonLin two-compartment model analysis from time zero to the 24-hour time point.

## Results and Discussion

### Method validation

#### Accuracy, precision, linearity and goodness of fit

To assess the exactness of an assay, the accuracy is measured through the repeated analysis of the sample. In this study, accuracy was determined through the measurement of three QC controls extrapolated from a standard curve. We express accuracy as the percent error or bias of ≤30% in serum and tissue homogenate. As we are qualifying this assay in a discovery setting, this accuracy goal is acceptable for that of an immunoassay^26^. Our second measure of assay robustness, precision, measures the repeatability expressed by the coefficient of variation (CV%) between QCs within a single plate (intra-assay precision) and between different plates (inter-assay precision) over separate assay days and between separate analysts performing the assays. We deemed that acceptable levels of precision for a discovery setting to be below 20%. Summaries of our accuracy and precision for both serum and liver tissue homogenate are shown in Table 1. Lastly, the “goodness of fit” is determined through the standard curve fit in the 4-PL model. The regression coefficient (R^2^) is an expression of the goodness of fit and as it approaches a value of 1, it is indicative of a good curve fit^26^. Calibration curve fits for the qualification runs of the POE immunoassay all had R^2^ values of 0.99 (curve parameters shown in Supplementary Information). Dilutional linearity for 1000-fold dilution in serum and 10,000-fold dilution in liver tissue homogenate was found to be %CV 3.4, −13.0%Bias and %CV 9.2, −9.0%Bias respectively, demonstrating acceptable dilutional linearity. Full calibration curve parameters and results by day are summarized for serum and tissue homogenate are summarized in Supplemental Tables 2 and 3.

**Table 1:** Accuracy and precision results within-day and between-days for the qualification of HPRT.siRNA antisense POE immunoassay in Sprague-Dawley serum and liver tissue homogenate.

Qualification parameters of HPRT-siRNA POE immunoassay in Sprague-Dawley serum (antisense strand)					Qualification parameters of HPRT-siRNA POE immunoassay in Sprague-Dawley liver homogenate (antisense strand)				
	Within-day (n = 18)		Between-day (n = 18)			Within-day (n = 18)		Between-day (n = 18)	
	%CV	%Bias	%CV	%Bias		%CV	%Bias	%CV	%Bias
LQC	7.0 to 13.8	−20.2 to 4.0	16.1	−5.8	LQC	3.0 to 19.6	−8.2 to 8.4	11.6	−2.1
MQC	1.0 to 19.3	−11.5 to 1.3	18.7	−4.6	MQC	6.0 to 32.8	−11.3 to 16.9	16.4	0.9
HQC	4.1 to 24.2	−18.4 to 24.8	25.7	0	HQC	12.0 to 29.6	−34.4 to 24.0	26.7	−3.2

### Sensitivity and specificity

**Figure 2:**
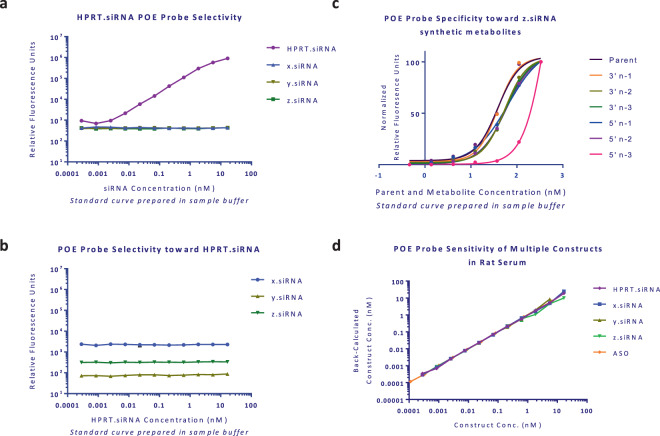
Assay selectivity of POE immunoassay probes holding capture and detection reagents constant with varying test article (**a**) and holding test article constant with varying detection reagents (**b**). Specificity of POE immunoassay probes against synthetic metabolites of z.siRNA (**c**).The assay is selective for 3′ and 5′ n-1 and n-2 metabolites with decreased reactivity with n-3 metabolites. EC_50_ values are shown in Supplementary Information. Normalized data represented with a nonlinear curve fit. Sensitivity of POE immunoassay probes for multiple constructs were assessed in rat serum with a dynamic range of 0.3 to 16,700 pM siRNA and 0.1 to 6,640 pM ASO (**d**). Mean data from A, B, and D shown with error bars representing standard deviation.

**Figure 3:**
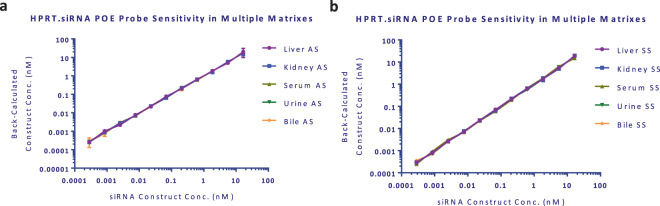
Calibration curve comparison in multiple Sprague Dawley matrixes for the antisense (**a**) and sense strand (**b**) versions of the POE immunoassay. Theoretical and back-calculated results were graphed in GraphPad Prism. Non-parametric Friedman test was performed using GraphPad Prism and showed no statistically significant difference at 5% significance level (p value = 0.956 and 0.847, respectively for **a,b**) demonstrating no changes in calibration curves generated in different matrices. Mean data is shown with error bars representing standard deviation.

To assess the sensitivity of the assay, standard curve spikes were prepared in their respective matrices across multiple species (Fig. 2d). We define the lower limit of detection as the lowest concentration of analyte that has a high probability of producing a response significantly greater than the response at the zero concentration. To assess the specificity of the assay, we assessed this in two parts: specificity of the probes to different metabolites (Fig. 2c) and specificity of the probes to different siRNA sequences (Fig. 2a,b). Shifts in EC_50_ values are summarized numerically in Supplemental Table 1. Two assays were performed to assess the specificity of the assay probes to different siRNA sequences. The first assay used HPRT-siRNA specific probes run against its respective sequence’s standard curve as well three additional standard curve spikes for siRNA compounds X, Y, and Z. The second assay flipped the detection output with HPRT-siRNA standard curves against the respective probes for siRNA compounds X, Y, and Z. To demonstrate assay performance in biological matrices, both sense and antisense strand were quantified out of standard curves prepared in Sprague Dawley liver and kidney homogenates, serum, urine, and bile (Fig. 3). Full sensitivity profiles for all siRNA constructs and ASO are summarized in Supplemental Figs. 1 and 2. m

### Stability of HPRT.siRNA

The POE immunoassay was applied to assess the stability of HPRT.siRNA antisense strand with repeated freeze thaws and with various temperature conditions. We found that our sample recovery remained consistent at all levels tested after three consecutive freeze thaws at room temperature. We also assessed the compound stability at room temperature for four hours and at −80 °C for greater than one week for both serum and liver tissue homogenates. We followed these parameters as they adhere to our general lab practices regarding study sample handling. We found the antisense strand of HPRT.siRNA to be stable under all conditions which are summarized in Table 2.

**Table 2:** Stability of HPRT.siRNA antisense strand assessed in Sprague-Dawley serum and liver homogenate with repeat freeze thaws and with storage at room temperature (4 hours) and deep freeze (−80 °C, ~2 weeks).

HPRT.siRNA Stability with Freeze Thaws (antisense strand)									
	Serum (n = 3)		Liver Homogenate (n = 3)						
	%CV	%Bias	%CV	%Bias					
LQC	7.7	−3.0	2.1	−0.7					
MQC	9.1	16.0	4.5	−10.5					
HQC	8.3	19.9	19.9	−4.0					
**HPRT.siRNA Stability at Room Temperature (antisense strand)**					**HPRT.siRNA Stability at −80 °C (antisense strand)**				
	**Serum (n = 3)**		**Liver Homogenate (n = 3)**			**Serum (n = 3)**		**Liver Homogenate (n = 3)**	
	%CV	%Bias	%CV	%Bias		%CV	%Bias	%CV	%Bias
LQC	3.6	−3.0	4.8	−14.0	LQC	6.5	5.0	2.8	−8.7
MQC	8.6	−2.6	4.5	−8.4	MQC	2.6	6.5	0.2	−3.5
HQC	4.3	6.4	12.3	−5.6	HQC	9.2	25.6	11.4	0.0

### Assay of HPRT.siRNA in a mixture

**Figure 4:**
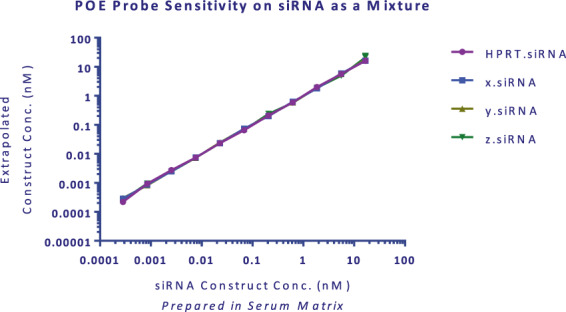
POE immunoassay probe selectivity to for the antisense strands of four unique siRNAs in a mixture. All siRNA molecules were added to a single calibration curve from 0.3 to 16,700 fM in Sprague Dawley serum. Independent probe sets were used to detect their corresponding antisense strands. Theoretical and back-calculated results were graphed in GraphPad Prism. Non-parametric Friedman test was performed using GraphPad Prism and showed no statistically significant difference at 5% significance level (p value = 0.983) demonstrating no changes in calibration curves when siRNAs prepared as a mixture. Mean data is shown with error bars representing standard deviation.

Robust standard curves were generated for both sense and antisense strands when four siRNA molecules were spiked into buffer as a mixture (Fig. 4). The assay standard curve range was linear for all strands and constructs from a tested assay range of 0.3 to 16,700 pM duplex siRNA. No interference among strands was observed for the individual siRNA molecules. From this data, we suggest that administration of a mixture of siRNAs or ASOs is capable of individual strand quantification with similar dynamic range and sensitivity to that of a single molecule. This expands the application of this assay for future therapies that may simultaneously administer unique sequences.

### Application *in vivo*

To demonstrate practical application of the POE immunoassay three siRNAs, unique in sequence, topology and/or chemical modifications, were administered subcutaneously to either male C57BL/6 mice at 5 mg/kg (HPRT.siRNA), or Sprague Dawley rats at a 10 mg/kg dose (x.siRNA and y.siRNA). In the Sprague Dawley rats, corresponding mRNA target sequences were not expressed, whereas HPRT mRNA was expressed in C57BL/6 mice.

**Figure 5:**
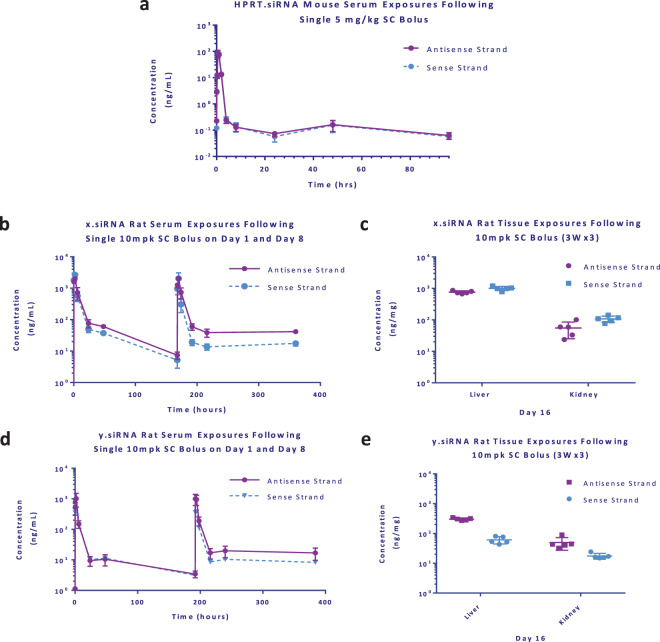
Serum concentration-time profile and tissue concentration of antisense and sense strands of HPRT.siRNA following a single SC bolus in C57BL/6 mice (**a**) 0–96 h in serum (n = 2 animals per time point). Serum concentration-time profile of x.siRNA (**b**) and y.siRNA (**d**) 0–48 h on day 1 and day 8 following a 10 mg/kg SC Bolus in male Sprague Dawley rats. Corresponding tissue concentrations of x.siRNA (**c**) and y.siRNA (**e**) 24 h post-dose following a QWx3 dosing regimen. Rat data plotted as individual replicates (n = 5 animals per time point). Mean data is shown with error bars representing standard deviation.

Serum concentration-time profiles were determined following a single dose of HPRT.siRNA for both sense and antisense strands (Fig. 5; Tables 3 & 4). Peak serum concentrations for HPRT.siRNA occurred at 0.5 hrs for the antisense strand, and 1 hr for the sense strand, with rapid clearance from the serum compartment as expected for a GalNAc-siRNA molecule^16,27^. A small population of antisense and sense strand remained in circulation for at least 96 hours post dose. A slight increase in the concentration of the sense and antisense strands was observed at 48 hrs. This uptick in the PK is a phenomenon we have observed in multiple studies across multiple siRNA molecules (data not shown), though the underlying mechanism is not well understood.

**Table 3:** Pharmacokinetic parameters of HPRT.siRNA antisense and sense strands in C57BL/6 mouse serum shown as Mean (SD). T_max_ = time of maximum concentration; AUC_last_ = area under the concentration time-curve from time 0 to last measurable concentration. All values are reported to 3 significant digits, with the exception of T_max_, which is reported as median value.

	HPRT.siRNA Pharmacokinetic Parameters in Serum		
	(n = 2)		
	T_max_ (hr)	C_max_ (ug/ml)	AUC_last_ (hr*ug/ml)
Antisense Strand	0.5	0.0830	0.120
Sense Strand	1	0.0822	0.125

**Table 4:** Pharmacokinetic parameters of x.siRNA and y.siRNA antisense and sense strands in Sprague Dawley rat serum shown as Mean (SD). T_max_ = time of maximum concentration; AUC_last_ = area under the concentration time-curve from time 0 to last measurable concentration. All values are reported to 3 significant digits, with the exception of T_max_, which is reported as median value.

	x.siRNA Pharmacokinetic Parameters in Serum						y.siRNA Pharmacokinetic Parameters in Serum					
	Day 0 (n = 5)			Day 7 (n = 5)			Day 0 (n = 5)			Day 7 (n = 5)		
	T_max_ (hr)	C_max_ (ug/ml)	AUC_last_ (hr*ug/ml)	T_max_ (hr)	C_max_ (ug/ml)	AUC_last_ (hr*ug/ml)	T_max_ (hr)	C_max_ (ug/ml)	AUC_last_ (hr*ug/ml)	T_max_ (hr)	C_max_ (ug/ml)	AUC_last_ (hr*ug/ml)
Antisense Strand	0.25	1.81	19.99	0.25	0.96	12.75	2	1.08	5.41	0.3	1.01	6.42
Sense Strand	2	2.05	17.73	2	2.04	16.98	2	0.47	3.62	2	0.37	3.00

Additionally, multiple dose pharmacokinetics was determined with x.siRNA and y.siRNA. Serum concentration-time profiles were determined for these unique siRNA molecules following three doses of 10 mg/kg once weekly with serum collections taken on day 0 and day 7 after subcutaneous dose (n = 5 animals per dosing group). Both x.siRNA and y.siRNA share a similar sequence and chemical modification pattern, yet differ in their topology with x.siRNA having two-nucleotide overhangs on both ends and y.siRNA having a single “blunted” end. In comparing the x.siRNA and y.siRNA rat PK data, we observed an AUC difference of 3.7-fold on day 0, followed by a 2-fold difference on day 7, with higher serum exposures for x.siRNA. We also observed differences in the AUC ratio of sense versus antisense strands, with the AUC ratio increasing for both molecules after the second dose. Reflecting the increased serum AUC of x.siRNA, there was also greater liver tissue distribution after three doses as compared to y.siRNA. Liver exposures (calculated means) for x.siRNA antisense and sense strands were measured to be 760 ng/mg and 1000 ng/mg, respectively. Kidney exposures were measured to be 55.3 ng/mg and 108 ng/mg, respectively. These exposure results suggest equivalent delivery and accumulation of both antisense and sense strands at the timepoint taken of 24 hours post third subcutaneous dose. The y.siRNA tissue exposures for antisense and sense strands were found to be 61.1 ng/mg and 303 ng/mg, respectively in the liver and 17.6 ng/mg and 50.0 ng/mg, respectively in the kidney. With regard to the antisense exposures between x.siRNA and y.siRNA, there was an approximate 12-fold increase in liver exposure of x.siRNA relative to y.siRNA.

## Conclusion

Improving upon methods we published previously^25,28^, the POE immunoassay demonstrates an ultrasensitive, extraction-free, enzyme-free format for the effective quantification of ASO and siRNA molecule in serum, tissue homogenates and excreta. Building upon the increased hybridization affinity and stability offered from locked-nucleic acid (LNA) modifications, we heavily modified our probes to improve stabilization of short sequences—removing the need for stability-driven probe extension and background signal reduction with enzymatic cleavage^29,30^. Assay sensitivity and dynamic range is amplified by electrochemiluminescent (ECL) readout through the Meso Scale Discovery (MSD) ECL platform. In our work with previous and re-tooled versions of our hybridization assays, we found that the removal of enzymatic reactions has improved assay reproducibility, throughput, and ease. In demonstrating the general applicability of this assay, we showcase three sequence-and structure-diverse siRNA molecules and one ASO. The capabilities of the POE immunoassay in measuring both sense and antisense strands of the siRNA duplex also proves valuable in the assessment of both strand and duplex stability as well as distribution kinetics of the two drug components. We also provide an example of assay robustness via a full qualification of the antisense strand of a representative siRNA.

RNAi through siRNA involves a complex mechanism of action involving the recruitment of multiple proteins, making the rate-determining step in therapeutic efficacy difficult to pinpoint. In addition, the unique biophysical properties of these molecules lead to atypical absorption, distribution, metabolism and elimination (ADME) processes, as well as the possibility of pharmacokinetics-pharmacodynamics (PKPD) uncoupling. These understated complexities make *in vitro* and *in vivo* effects for these therapeutics challenging. Lacking this understanding leads to inefficiencies in translation throughout the drug development process, including increased animal usage, and may lead to an elevated risk in first in human trials. The first step forward in correcting this discrepancy is to establish a robust bioanalytical toolbox to understand these relationships. The author’s intention is for this assay to be the gold standard in quantification of oligonucleotide therapeutics in the field. We further stress the importance of the measurement of both strands as they are key in understanding the stability and metabolism pathways of siRNA therapeutics. Understanding the strand kinetic relationships are key to developing a mechanistic PKPD model to elucidate the rate limiting step in the pharmacology of RNA inhibition, aiding in the design of new molecular entities.

## Supplementary information

Supplementary Information.
