# Supplementary material for: POE Immunoassay: Plate-based oligonucleotide electro-chemiluminescent immunoassay for the quantification of nucleic acids in biological matrices
Source: Sci Rep. 2020 Jun 26;10:10425. doi: 10.1038/s41598-020-66829-6 (PMC7319975; doi:10.1038/s41598-020-66829-6)
Supplement: Supplementary file 1 — Supplementary Information. [file 41598_2020_66829_MOESM1_ESM.pdf]

Supplemental Information

POE Immunoassay: Plate-based oligonucleotide electro-chemiluminescent immunoassay for the quantification of nucleic acids in biological matrixes.

Mai B. Thayer[1,], Sara C. Humphreys[1], Julie M. Lade[1], Kyu S. Chung[1], and Brooke Rock[1,*]

[1] Amgen Research, Pharmacokinetics & Drug Metabolism, Amgen Inc., South San Francisco, CA

* To whom correspondence should be addressed.

*Brooke M. Rock, Tel: 650 244-2845, Email: brooke@amgen.com.

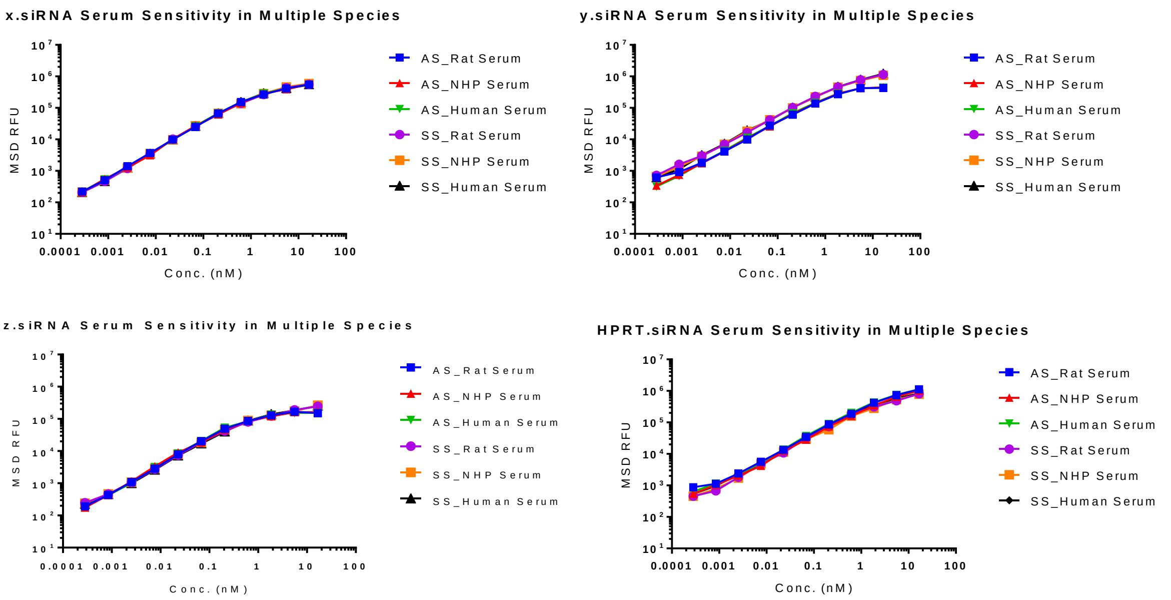

Supplemental Figure 1. Sensitivity of siRNA constructs in Sprague Dawley, non-human primate (NHP), and human serum. Sense and antisense strands detected independently with the POE immunoassay.

	Parent	3' n-1	3' n-2	3' n-3	5' n-1	5' n-2	5' n-3	n-2	n-4	n-6
**%Reactivity from parent (calculated from EC50)**	100	99.8	70.3	67.7	60.2	65.2	18.4	96.5	73.0	0

Supplemental Table 1. Selectivity of z.siRNA POE immunoassay probes to synthetic metabolites. EC50 calculated with a 4-parameter fit with fixed weighting in GraphPad Prism Software 7.02 (La Jolla, CA). N-x(metabolites) signify an exclusion from both ends of the siRNA strand. For example n-2 corresponds to one base removed from both the 5′ and 3′ ends.

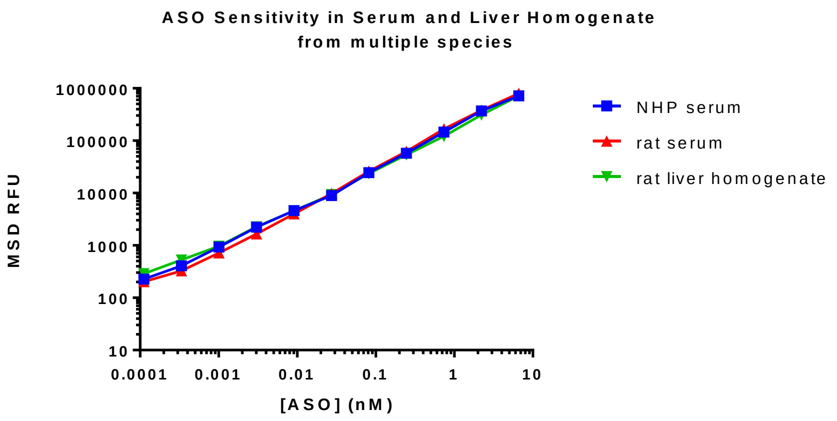

Supplemental Figure 2. Sensitivity of ASO construct in NHP serum and Sprague Dawley serum and liver tissue homogenate.

Calibration Curve Parameters for HPRT.siRNA Calibration Standards in Rat Serum, analysis of antisense strand (Study POE Immunoassay MSD assay qualification)									
Run Date	Curve Number	Min	Max	Slope	Ed50	R-Squared	LLOQ	ULOQ	Regression Footnote(s)
Day1	1	498	764000	1.01	1140	0.9985	4.00000	2500.00	1
Day2	2	-119	1360000	0.949	1690	0.9994	4.00000	2500.00	1
Day2	3	690	1980000	0.976	1610	0.9989	4.00000	2500.00	1
Day3	4	211	894000	0.978	1090	0.9992	4.00000	2500.00	1
Day3	5	325	1440000	0.971	1790	0.9982	4.00000	2500.00	1
Day4	6	401	789000	0.963	988	0.9980	4.00000	2500.00	1
Day4	11	455	1050000	0.981	893	0.9977	4.00000	2500.00	1
Day5	12	435	1180000	0.983	1070	0.9977	4.00000	2500.00	1
Day5	13	-160	1220000	0.948	1320	0.9986	4.00000	2500.00	1
Day6	21	697	1700000	1.00	962	0.9984	4.00000	2500.00	1
Mean		343	1237700	0.976	1255	0.9985			
S.D.		294.075	394876	0.02	328	0.00059			
%CV		85.6613	31.904	2.026	26.1	0.05863			
n		10	10	10	10	10			
Regression Footnote(s):									
1) Resp. = (Min - Max) / (1 + (Conc. / Ed50) ** Slope) + Max									

Supplemental Table 2. Calibration curve parameters for the qualification runs of the antisense strand of HPRT.siRNA in Sprague Dawley

Calibration Curve Parameters for HPRT.siRNA Calibration Standards in Rat Liver Homogenare, analysis of antisense strand (Study POE Immunoassay MSD assay qualification)									
Run Date	Curve Number	Min	Max	Slope	Ed50	R-Squared	LLOQ	ULOQ	Regression Footnote(s)
Day1	15	-696	975000	0.902	1480	0.9969	4.00000	2500.00	1
Day1	16	-88.7	892000	0.909	1390	0.9972	4.00000	2500.00	1
Day1	17	-228	1600000	1.01	1510	0.9962	4.00000	2500.00	1
Day1	18	-2.66	1910000	0.941	1710	0.9985	4.00000	2500.00	1
Day1	19	1010	1820000	0.976	918	0.9982	4.00000	2500.00	1
Day1	20	1730	1820000	0.979	1230	0.9974	4.00000	2500.00	1
Day2	22	607	1800000	0.988	1180	0.9992	4.00000	2500.00	1
Day3	24	475	1390000	1.01	836	0.9992	4.00000	2500.00	1
Day3	27	501	1680000	1.02	1230	0.9996	4.00000	2500.00	1
Mean		368	1543000	1	1276	1			
S.D.		723	378492	0.044	281	0.00118			
%CV		197	24.5296	4.509	22	0.11835			
n		9	9	9	9	9			
Regression Footnote(s):									
1) Resp. = (Min - Max) / (1 + (Conc. / Ed50) ** Slope) + Max									

Supplemental Table 3. Calibration curve parameters for the qualification runs of the antisense strand of HPRT.siRNA in Sprague Dawley liver tissue homogenate.

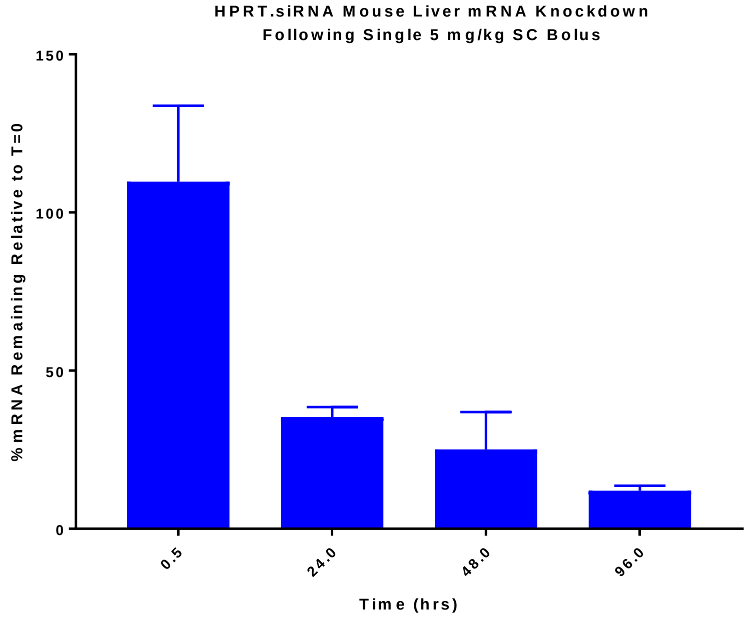

Supplemental Figure 3. Efficacy of HPRT.siRNA demonstrated as percentage of knockdown relative to T=0 liver samples in mouse liver samples. In short, samples were homogenized, and RNA extracted using a MagMax^{TM}-96 Total RNA Isolation Kit (Thermofisher, AM1830). Reverse transcript was generated using AccuScript High Fidelity RT-PCR System (Agilent, 600180 and samples were analyzed by on a Digital Droplet PCR system (BioRad, USA) using TaqMan Primer Probe FAM/HEX probes: Mouse HPRT TaqMan FAM-MGB (Thermofisher, 4331182) and mouse TBP TaqMan VIC-MGB (Thermofisher, 4448489). Results normalized to TBP housekeeping gene.
